# Immediate and Delayed Implant Placement Protocols: A Comparative Case-Based Perspective

**DOI:** 10.7759/cureus.107660

**Published:** 2026-04-24

**Authors:** Abdul Habeeb Bin Mohsin, Mandapathi Priyanka, Mahitha Gogu, Jyotsna Amalakara, Venkata Aruna

**Affiliations:** 1 Department of Prosthodontics and Implantology, Coral Dental and Implants Centre, Hyderabad, IND; 2 Department of Periodontology, Smile Central Dental Clinic, Hyderabad, IND; 3 Department of Health Informatics, Indiana University Indianapolis, Indianapolis, USA; 4 Department of Periodontology, Mamata Institute of Dental Sciences, Hyderabad, IND

**Keywords:** delayed implant placement, dental implants, immediate implant placement, immediate loading, osseointegration

## Abstract

Dental implants have become a predictable and widely accepted treatment modality for replacing missing teeth, offering functional and esthetic rehabilitation. Advancements in implant design and surgical protocols have enabled clinicians to adopt various placement and loading strategies that are tailored to individual clinical scenarios. The selection of an appropriate protocol is critical for optimizing the treatment outcomes. This report describes two clinical scenarios involving a 55-year-old female patient who required mandibular first molar replacements. In the first scenario, the tooth was extracted atraumatically, followed by immediate implant placement; loading was deferred until after a six-month healing period. In the second scenario, implant placement was postponed for six months post-extraction, after which immediate nonfunctional loading was carried out within 48 hours. Standardized surgical protocols were followed in both cases to ensure adequate primary stability and preservation of the surrounding tissues. Clinical and radiographic evaluations revealed successful osseointegration, stable peri-implant soft tissues, and minimal marginal bone loss using both approaches. No complications, such as infection, mobility, or peri-implant inflammation, were observed during follow-up. Within the limitations of this report, immediate implant placement with delayed loading and delayed implant placement with immediate nonfunctional loading demonstrated predictable and favorable outcomes when appropriate case selection and clinical protocols were followed.

## Introduction

Dental implants have revolutionized contemporary prosthetic dentistry by providing predictable and functionally stable solutions for the replacement of missing teeth. Unlike conventional fixed dental prostheses, implant-supported restorations allow for preservation of the adjacent tooth structure while closely mimicking the biomechanics of natural teeth [1]. Implant primary stability is a critical determinant of successful osseointegration and is influenced by surgical techniques and bone characteristics, as demonstrated by Agarwal et al. [2], who reported improved stability outcomes with osseodensification techniques.

Advancements in implant design, surface modifications, and surgical techniques have led to the development of alternative protocols, including immediate implant placement and immediate or early loading strategies. Immediate implant placement, performed at the time of tooth extraction, offers several clinical advantages such as preservation of alveolar bone architecture, reduced treatment duration, and fewer surgical interventions [3]. On the other hand, delayed implant placement allows for complete soft and hard tissue healing, potentially improving implant positioning and stability in certain clinical scenarios [4].

Similarly, the loading protocols underwent significant transformations. Immediate loading, particularly non-functional loading, has gained acceptance owing to its ability to enhance patient comfort and esthetics by reducing edentulous periods [5]. However, careful case selection, primary stability, and controlled occlusal loading remain the critical determinants of success. Despite these advancements, the choice between immediate and delayed implant placement and loading protocols remains dependent on multiple factors, including bone quality, soft tissue conditions, and clinician expertise [5,6]. The aim of this report was to present and compare two clinical cases involving mandibular first molar replacement using immediate implant placement with delayed loading and delayed implant placement with immediate non-functional loading, and to evaluate their clinical outcomes in terms of implant stability and peri-implant tissue response.

## Case presentation

Case scenario 1

**Figure 1:**
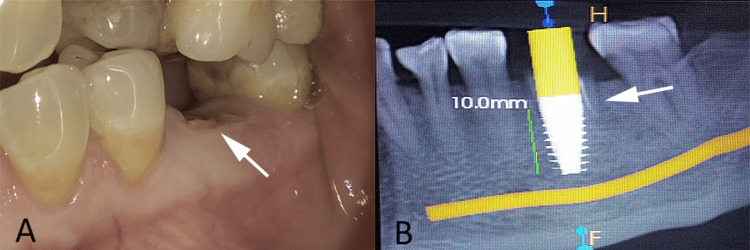
Clinical and radiographic evaluation findings A: clinical view showing a grossly decayed mandibular left first molar with residual root stumps (white arrow); B: preoperative radiograph demonstrating adequate interradicular and apical bone for immediate implant placement (white arrow). Original patient images, used with the patient's permission.

A 55-year-old female patient presented with the chief complaint of pain and difficulty in mastication in the left posterior mandibular region. Her medical history was non-contributory, with no history of systemic illness or smoking. Dental history revealed progressive deterioration of the mandibular left first molar with no prior successful restorative intervention. On clinical examination, the mandibular left first molar was grossly decayed with residual root stumps and was deemed non-restorable (Figure 1A). The surrounding gingival tissues were healthy, with no signs of inflammation or suppuration, and the gingival biotype was favorable for implant therapy. Preoperative radiographic evaluation revealed adequate interradicular bone width and sufficient apical bone to achieve primary stability (Figure 1B).

**Figure 2:**
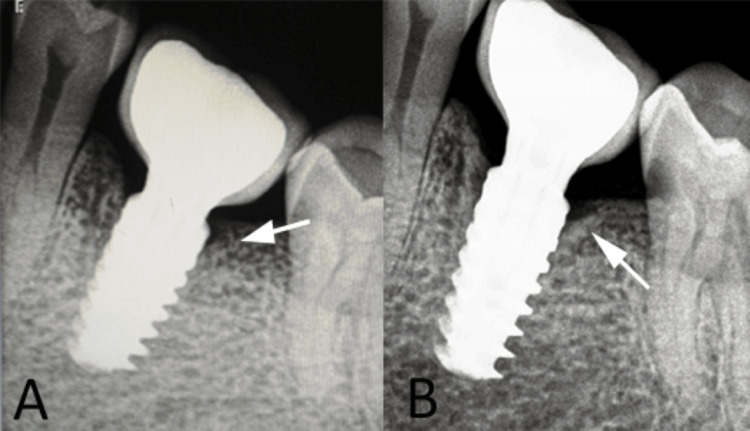
Follow-up evaluation findings A: post-loading radiograph showing crestal bone levels around the immediate implant (white arrow); B: three-year follow-up radiograph demonstrating minimal to mild marginal bone loss (white arrow). Original patient images, used with the patient's permission.

Preoperatively, the patient received prophylactic medication and was instructed to rinse with 0.12% chlorhexidine digluconate solution (Hexidine®, ICPA Health Products Ltd., Ankleshwar, Gujarat, India) for 30-60 seconds. Under local anesthesia, atraumatic extraction was performed using minimally invasive techniques to preserve the socket architecture and buccal cortical plate. Immediate implant placement was performed following osteotomy preparation according to standard drilling protocols. A root-form titanium implant (Touareg™ S Implant, ADIN Dental Implant Systems) was inserted into the extraction socket, achieving satisfactory primary stability. No provisional restoration was provided, and the implant was left unloaded to allow undisturbed osseointegration. After a healing period of six months, a second-stage surgery was performed, and a healing abutment was placed to facilitate soft tissue contouring. Subsequently, impressions were made using polyvinyl siloxane material (Aquasil®, Dentsply Sirona, Charlotte, North Carolina, USA), and a definitive screw-retained prosthesis was fabricated and delivered. Follow-up clinical and radiographic evaluation demonstrated excellent soft tissue adaptation, absence of pain or infection, and stable peri-implant conditions with minimal to mild marginal bone loss, indicating successful implant integration (Figure 2).

Case scenario 2

**Figure 3:**
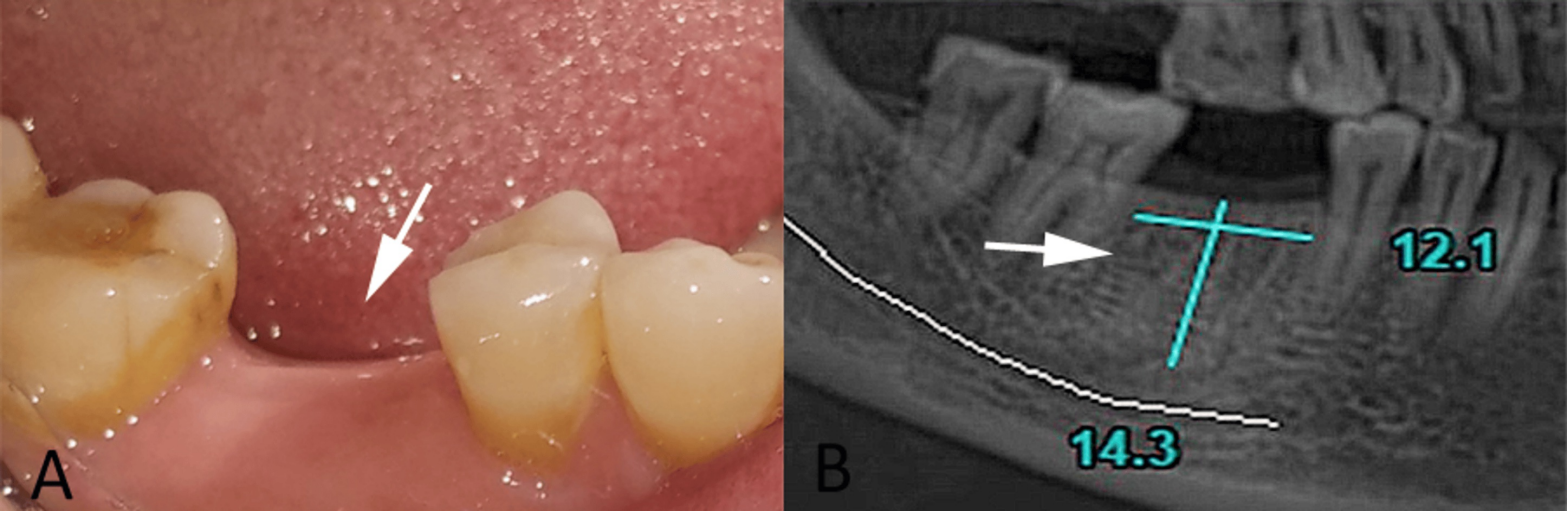
Clinical and radiographic evaluation findings A: clinical view showing edentulous space in the mandibular right first molar region (white arrow); B: preoperative radiograph demonstrating adequate bone dimensions for delayed implant placement (white arrow). Original patient images, used with the patient's permission.

The same 55-year-old female patient presented one year later with a complaint of a missing tooth in the mandibular right posterior region affecting mastication (Figure 3A). Her medical and dental histories remained unchanged, with no systemic risk factors or contraindications for implant therapy. Clinical examination revealed an edentulous space in relation to the mandibular right first molar, with a well-healed extraction site and healthy surrounding soft tissues. The gingival biotype was favorable for implant therapy. Radiographic evaluation demonstrated adequate bone height and width and appropriate interradicular space, indicating suitability for implant placement without the need for augmentation (Figure 3B).

**Figure 4:**
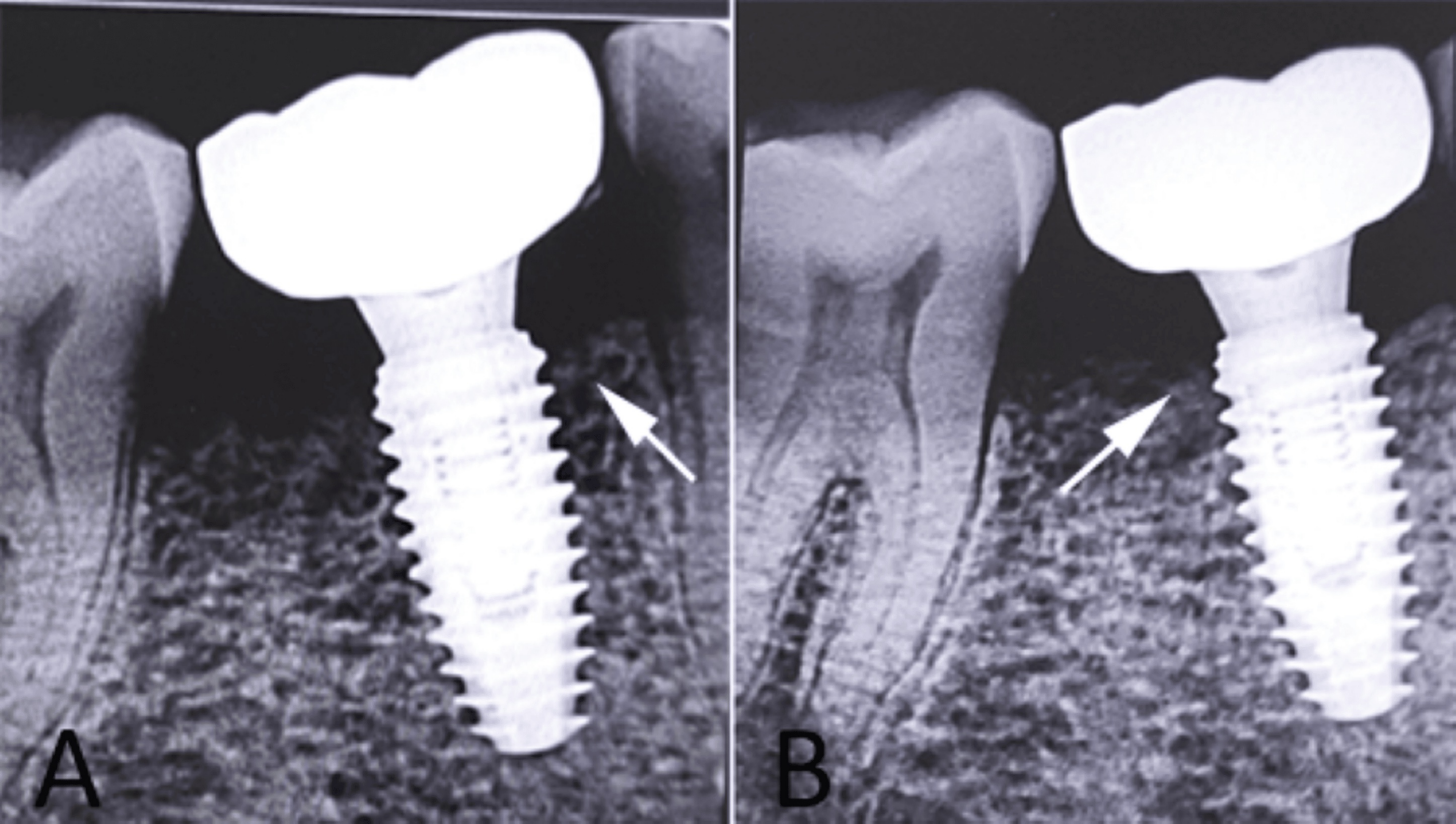
Follow-up evaluation findings A: post-loading radiograph showing stable crestal bone levels around the delayed implant (white arrow); B: three-year follow-up radiograph demonstrating maintained peri-implant bone levels (white arrow). Original patient images, used with the patient's permission.

Following standard preoperative protocol, including a 0.12% chlorhexidine mouth rinse, implant placement was planned after a healing period of six months post-extraction. After confirming satisfactory healing, implant placement was performed using a minimally invasive flapless punch technique. A root-form implant (IS II Active implant, Neobiotech) was inserted with good primary stability. Immediately following implant placement, impressions were made using polyvinyl siloxane material (Express™, 3M, Saint Paul, Minnesota, USA). Within 48 hours, a provisional crown was fabricated using auto-polymerizing acrylic resin and delivered under non-functional loading conditions. Care was taken to eliminate occlusal contacts in both centric and eccentric movements to prevent micromovement during the healing phase. After three months, the provisional restoration was replaced with a definitive cement-retained prosthesis. Follow-up clinical and radiographic evaluation demonstrated healthy peri-implant soft tissues, absence of inflammation or mobility, and stable crestal bone levels over time, confirming a successful treatment outcome (Figure 4).

## Discussion

The present report describes two clinical scenarios involving the same patient, both of which involve mandibular first molar replacement using different implant placement and loading protocols: immediate implant placement with delayed loading (Case 1) and delayed implant placement with immediate non-functional loading (Case 2). Both approaches demonstrated favorable clinical and radiographic outcomes with satisfactory implant stability, healthy peri-implant soft tissues, and minimal marginal bone loss over the follow-up period. The use of a single patient eliminated inter-individual variability, allowing for a more reliable comparison between the two protocols.

Immediate implant placement, as performed in Case 1, has been widely advocated for its ability to preserve alveolar bone architecture and reduce the overall treatment time. Studies by Quirynen et al. [7] and Botticelli et al. [8] demonstrated that implants placed in fresh extraction sockets show high survival rates and favorable bone preservation. In the present case, atraumatic extraction and preservation of the buccal plate contributed to the successful implant integration. The decision to delay loading allows undisturbed osseointegration, minimizing the risk of micromovement, which has been shown to adversely affect bone healing when exceeding critical thresholds. Atieh et al. [9] reported that immediate restoration/loading of single implants can be a predictable and effective approach when appropriate case selection and primary stability are achieved.

In contrast, Case 2 involved delayed implant placement following socket healing combined with immediate nonfunctional loading. This approach allows natural bone remodeling and maturation of soft tissues prior to implant placement, which may improve implant positioning and reduce the risk of peri-implant defects. Narang et al. [10] demonstrated that placement of multiple implants with immediate loading can achieve successful clinical outcomes when adequate primary stability and occlusal control are ensured. Vogl et al. [11] reported that both immediate occlusal and nonocclusal loading protocols showed comparable implant survival rates over 36 months. However, nonocclusal loading demonstrated a more favorable healing environment during the early phase, supporting its use in situations where a controlled load distribution is desired. In the present case, careful elimination of the occlusal contacts ensured that functional loading was avoided during the critical healing phase, thereby supporting successful osseointegration.

Comparatively, the two protocols resulted in similar outcomes in terms of implant survival and peri-implant tissue health. These findings are consistent with previous reports suggesting that when proper case selection and surgical principles are followed, both immediate and delayed approaches can yield predictable results [12,13]. In both cases presented here, adequate primary stability was achieved, which likely contributed to the favorable results observed. Felice et al. [14] reported that immediate non-occlusal loading of post-extractive implants demonstrated short-term outcomes comparable to those of delayed implant placement in preserved sockets. Their findings suggest that with proper case selection, immediate protocols can achieve predictable results without compromising implant success.

Singh et al. [15] presented a case demonstrating immediate implant placement with immediate loading following the extraction of natural teeth, highlighting successful clinical outcomes in terms of primary stability, osseointegration, and peri-implant tissue health. The authors emphasized that careful case selection, atraumatic extraction, and achievement of adequate primary stability are critical prerequisites for the success of immediate loading protocols. Their findings support the concept that immediate implant placement combined with immediate loading can reduce treatment time and improve patient satisfaction, although the study was limited to a single protocol without comparison to delayed approaches, unlike the present report, which evaluated two different strategies within the same patient.

From a clinical perspective, the choice between immediate and delayed implant placement should be guided by factors such as bone quality, soft tissue condition, presence of infection, and the clinician’s expertise. Immediate placement with delayed loading may be preferred in situations where preservation of alveolar architecture is critical, whereas delayed placement with immediate nonfunctional loading may be advantageous in cases requiring enhanced soft tissue healing and early esthetic rehabilitation.

Both treatment approaches demonstrated predictable outcomes, indicating that clinicians can tailor implant protocols according to individual case requirements. Immediate implant placement reduces treatment time and preserves bone, whereas delayed placement allows better tissue maturation and flexibility in prosthetic planning. Achieving primary stability and controlling occlusal loading remain key determinants of success. This report is limited by its nature as a two-case presentation and the absence of standardized radiographic measurements. The findings cannot be generalized because of the small sample size and the lack of long-term quantitative analysis. Further studies with larger cohorts and controlled designs are needed to validate these observations.

This study is limited by the absence of quantitative measurements for key clinical parameters. Primary stability was not assessed using objective metrics such as insertion torque (Ncm) or implant stability quotient (ISQ). Additionally, marginal bone loss was evaluated qualitatively without the use of standardized radiographic software to obtain precise linear measurements (e.g., from implant platform to first bone-to-implant contact). The lack of defined occlusal clearance in non-functional loading further limits reproducibility. Future studies incorporating these objective and standardized assessments are recommended to enhance accuracy and comparability.

## Conclusions

Both treatment approaches demonstrated favorable clinical and radiographic outcomes with successful osseointegration, stable peri-implant tissues, and minimal marginal bone loss. Immediate implant placement with delayed loading preserved the alveolar architecture, while delayed placement with immediate non-functional loading allowed satisfactory tissue healing and early restoration. Within the limitations of this case report, both protocols appear reliable and effective. Careful case selection, atraumatic surgical techniques, achievement of primary stability, and controlled loading are essential factors for ensuring predictable implant success.
